# Supplementary material for: Disruption of P2Y2 Signaling Promotes Breast Tumor Cell Dissemination by Reducing ATP-Dependent Calcium Elevation and Actin Localization to Cell Junctions
Source: Int J Mol Sci. 2025 May 1;26(9):4286. doi: 10.3390/ijms26094286 (PMC12071985; doi:10.3390/ijms26094286)
Supplement: Supplementary file 1 [file ijms-26-04286-s001.zip › IJMS_supplement_fullimages_MMull_032825.pdf]

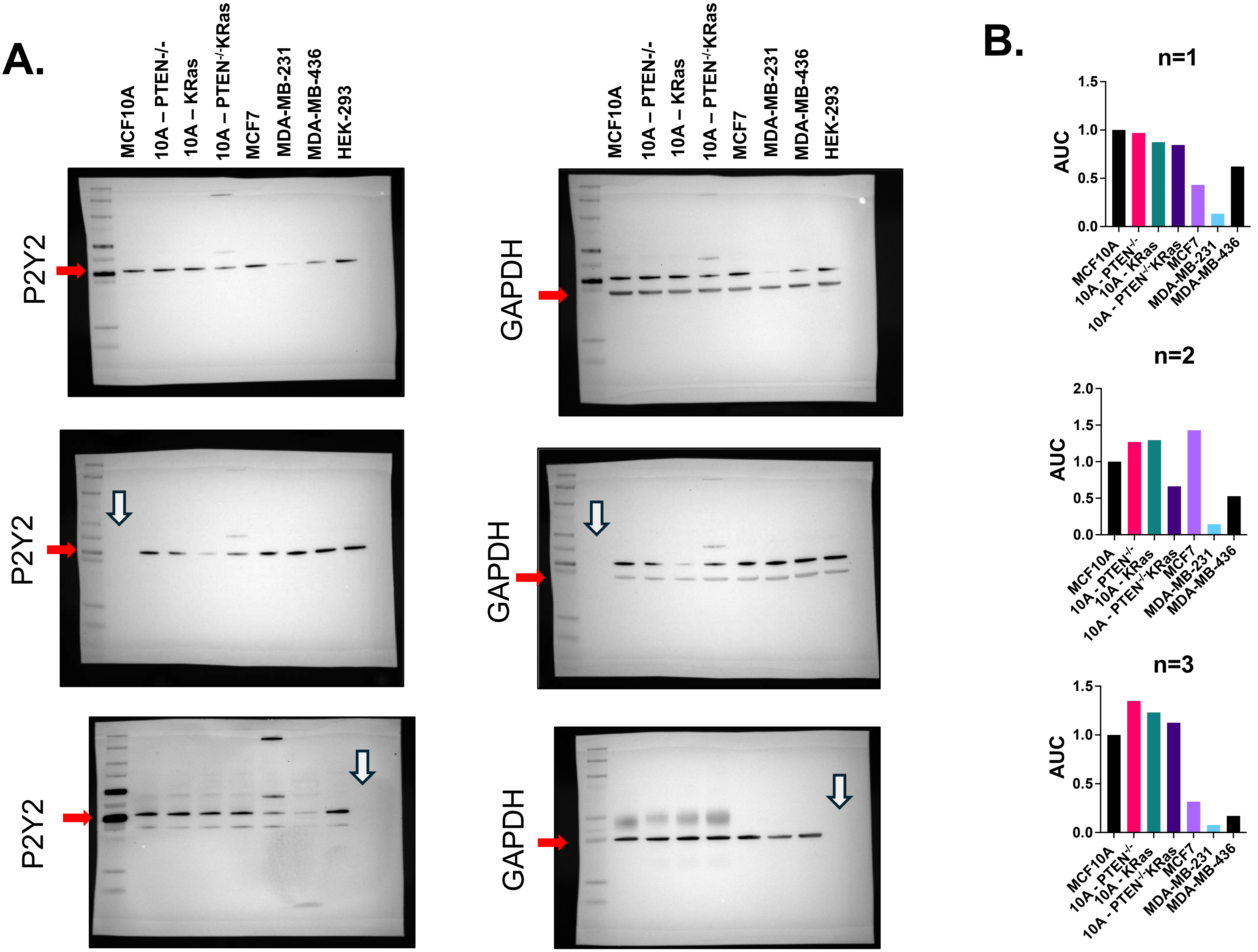
A.
MCF10A
10A – PTEN-/-
10A – KRas
10A – PTEN-/-KRas
MCF7
MDA-MB-231
MDA-MB-436
HEK-293
P2Y2
GAPDH
B.
n=1
n=2
n=3
AUC
MCF10A
10A - PTEN-/-
10A - KRas
10A - PTEN-/-KRas
MCF7
MDA-MB-231
MDA-MB-436

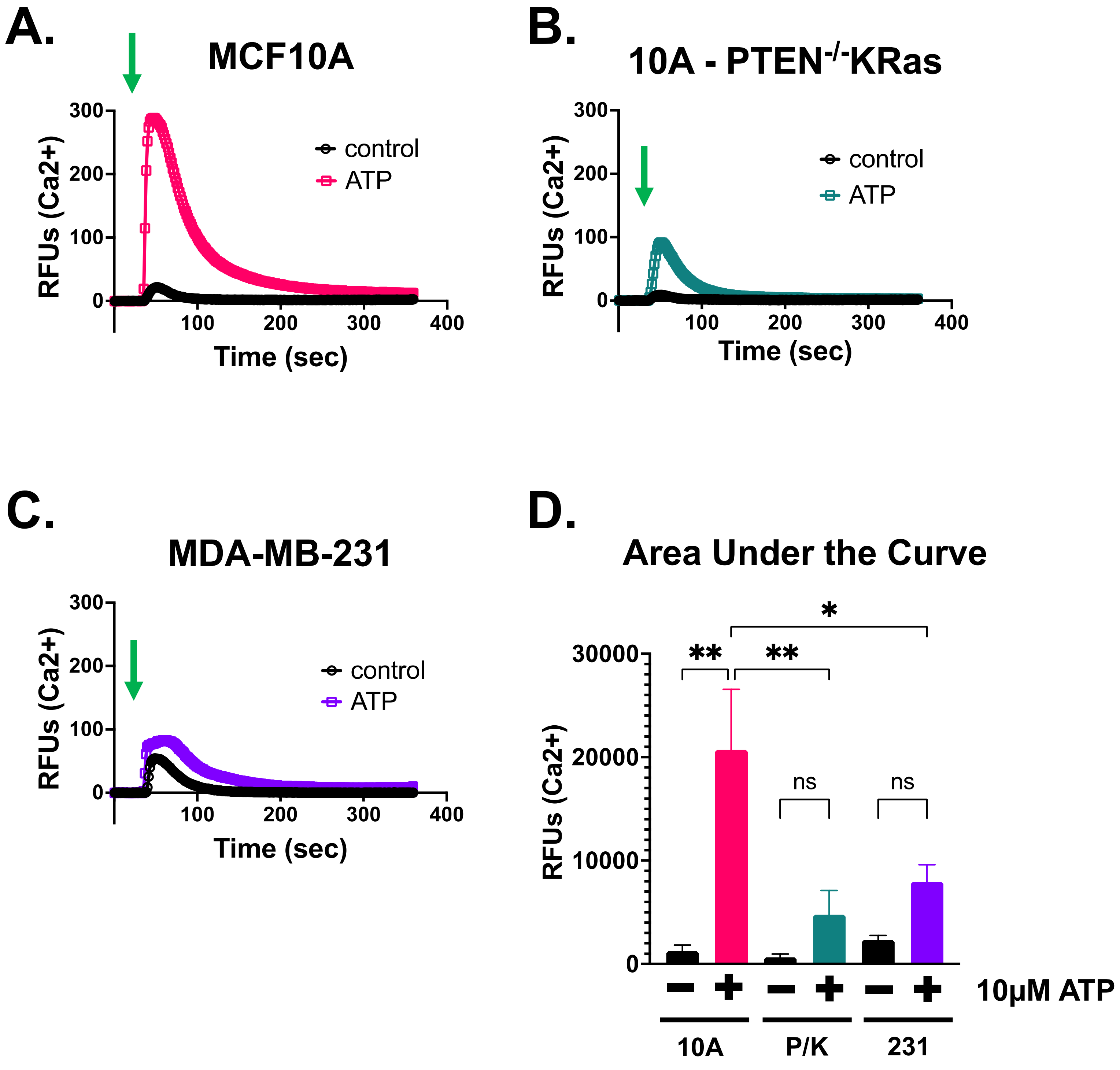
A.
MCF10A
RFUs (Ca2+)
Time (sec)
control
ATP
B.
10A - PTEN-/-KRas
RFUs (Ca2+)
Time (sec)
control
ATP
C.
MDA-MB-231
RFUs (Ca2+)
Time (sec)
control
ATP
D.
Area Under the Curve
RFUs (Ca2+)
**
**
*
ns
ns
10μM ATP
10A
P/K
231

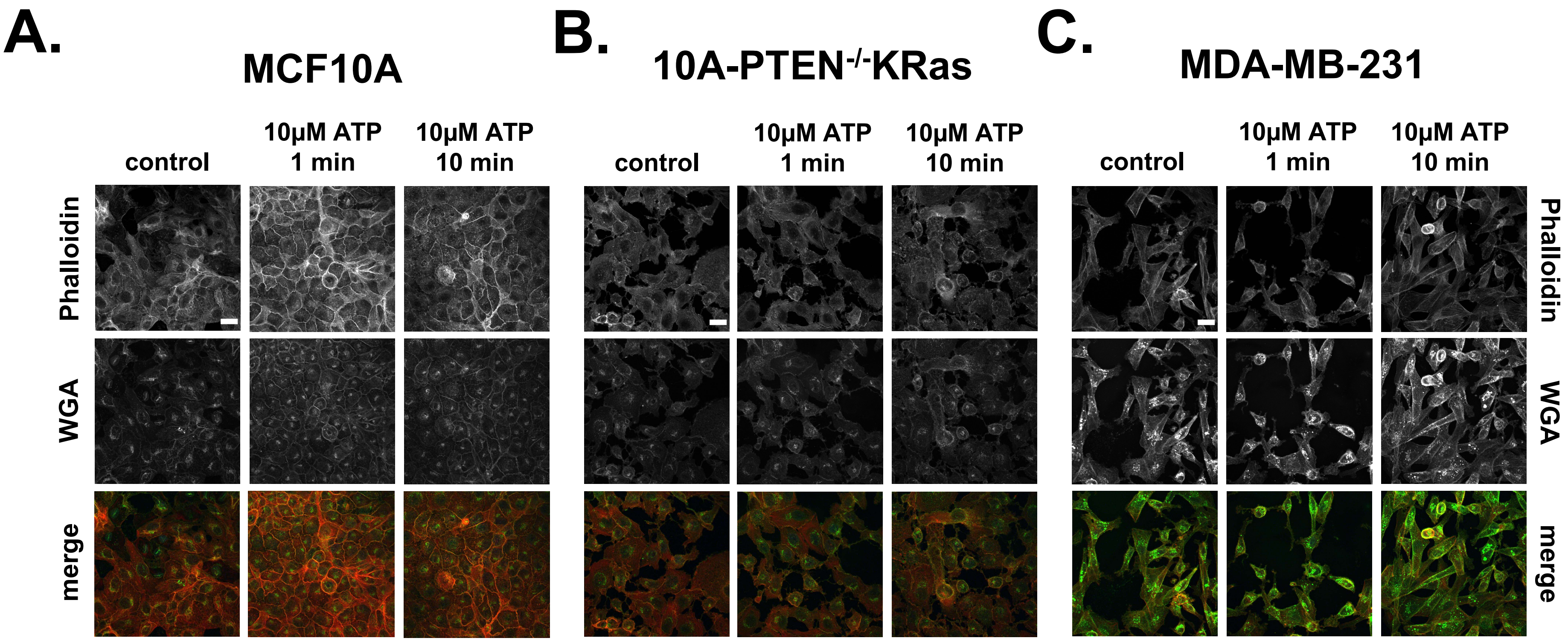

A.
MCF10A
B.
10A-PTEN-/-KRas
C.
MDA-MB-231
control
10μM ATP 1 min
10μM ATP 10 min
control
10μM ATP 1 min
10μM ATP 10 min
control
10μM ATP 1 min
10μM ATP 10 min
Phalloidin
WGA
merge
Phalloidin
WGA
merge

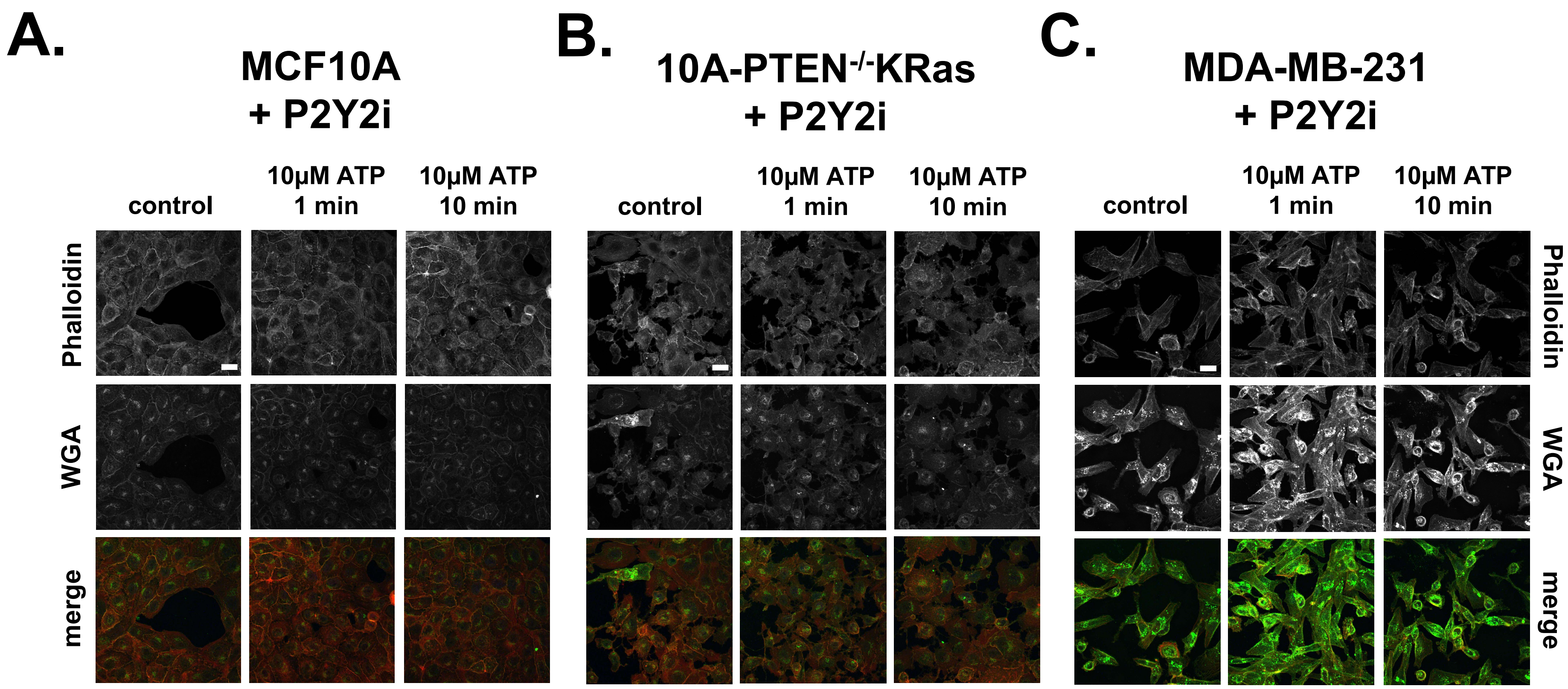
A.
MCF10A
+ P2Y2i
B.
10A-PTEN-/-KRas
+ P2Y2i
C.
MDA-MB-231
+ P2Y2i
control
10μM ATP
1 min
10μM ATP
10 min
Phalloidin
WGA
merge

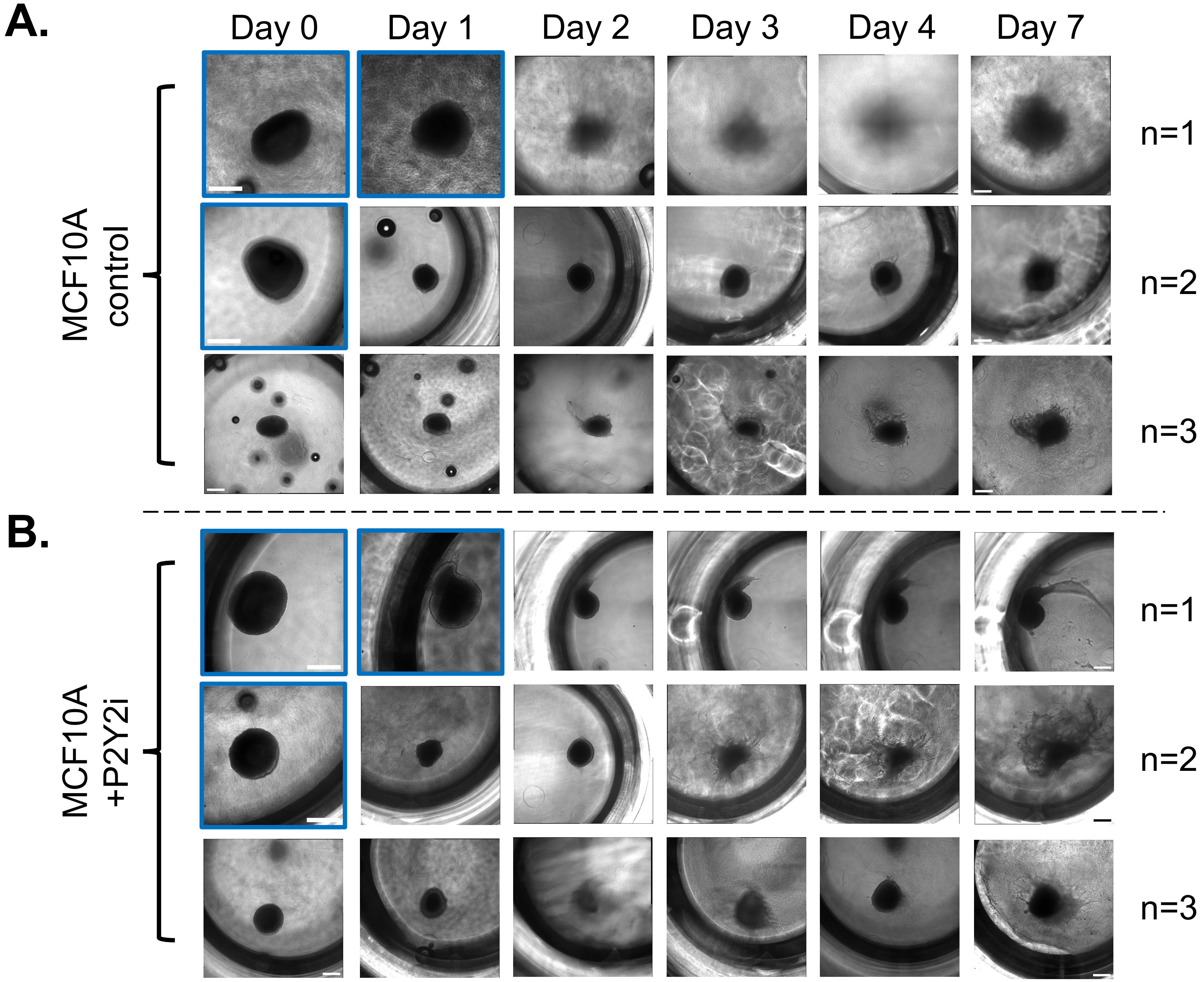
A.
Day 0
Day 1
Day 2
Day 3
Day 4
Day 7
MCF10A
control
n=1
n=2
n=3
B.
MCF10A
+P2Y2i
n=1
n=2
n=3

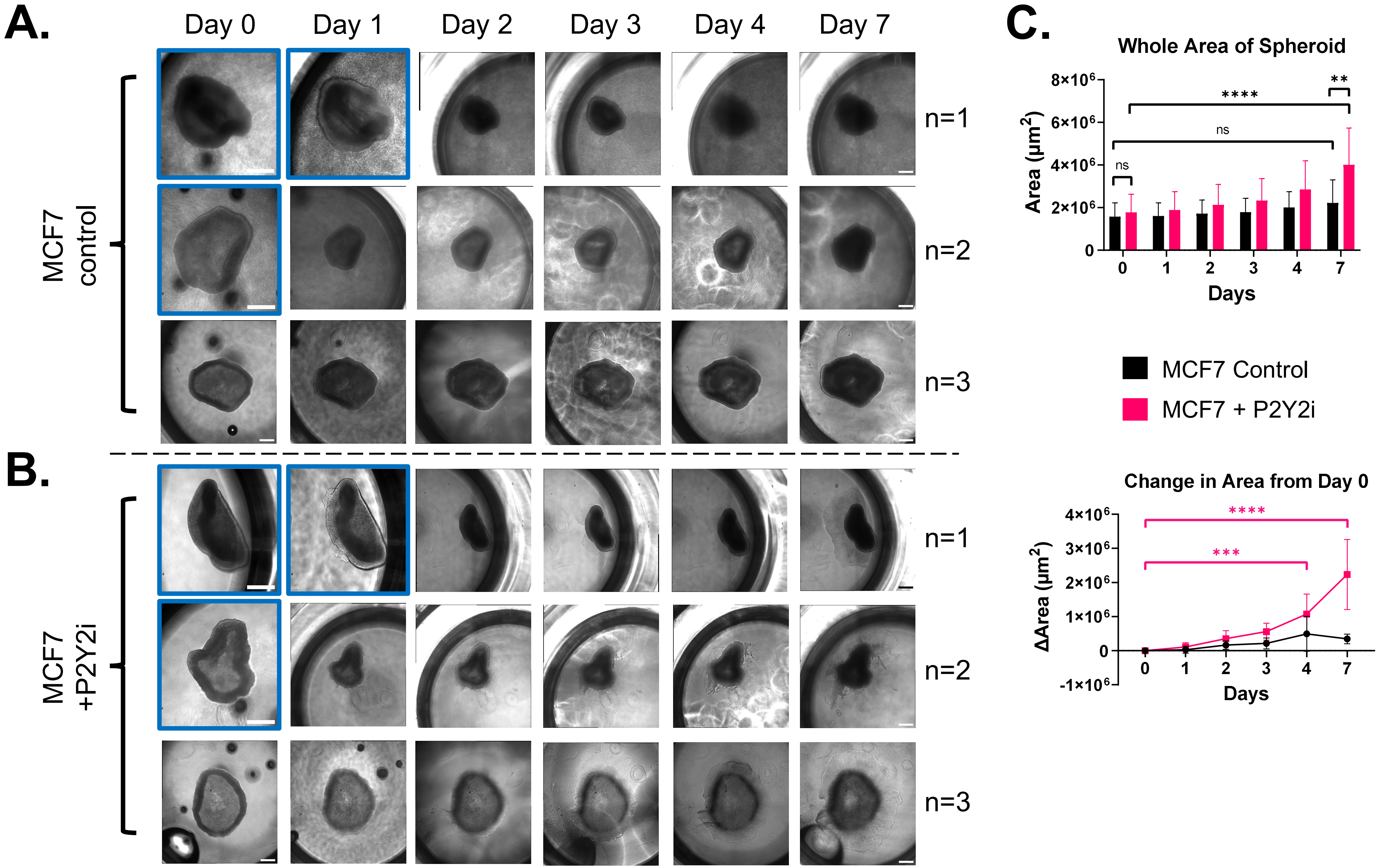
A.
Day 0
Day 1
Day 2
Day 3
Day 4
Day 7
MCF7 control
n=1
n=2
n=3
B.
MCF7 +P2Y2i
n=1
n=2
n=3
C.
Whole Area of Spheroid
Area (μm²)
8×10⁶
6×10⁶
4×10⁶
2×10⁶
0
ns
ns
****
**
0
1
2
3
4
7
Days
MCF7 Control
MCF7 + P2Y2i
Change in Area from Day 0
ΔArea (μm²)
4×10⁶
3×10⁶
2×10⁶
1×10⁶
0
-1×10⁶
****
***
Days
